# Navigating the Diagnostic Challenge of Aspergillus Spinal Epidural Abscess in an Immunocompetent Patient: A Case Report and Literature Review

**DOI:** 10.7759/cureus.42770

**Published:** 2023-07-31

**Authors:** Kunal Karmilkar, Aditi Patel, Troy M Vaughn

**Affiliations:** 1 Medicine, Edward Via College of Osteopathic Medicine (VCOM) - Louisiana, Monroe, USA; 2 Neurological Surgery, Alexandria Neurosurgical Clinic, Alexandria, USA

**Keywords:** infectious disease, neurosurgery, aspergillus abscess, spinal abscess, aspergillus spinal epidural abscess

## Abstract

Aspergillus spinal epidural abscess (ASEA) is a rare, life-threatening condition that can cause spinal cord compression with neurologic deficits. The diagnosis of ASEA can be challenging due to the atypical clinical presentation and low prevalence. We describe the successful management of a rare, immunocompetent, 85-year-old male with ASEA at the T12-L1 and L1-L2 levels and present a review of the literature. Based on most case reports and our knowledge, this is a rare presentation of ASEA in a patient without systemic symptoms, leukocytosis, or a history of immunosuppressive status due to chronic steroid use. The patient presented with multiple falls and lower extremity paraparesis with near-complete paralysis of the right lower extremity for a duration of three months. Systemic symptoms of infection were absent and standard lab evaluations were unremarkable. CT imaging identified cord signal changes at the level of T10-T11 and a contrast block at L1 suspicious for spinal stenosis and impingement. During lumbar spine exploration, purulent fluid consistent with an abscess was found in the epidural space. Cultures were forwarded to microbiology and returned with Aspergillus. Postoperatively, Infectious Disease (ID) recommended treatment with voriconazole, cefepime, and vancomycin, which yielded gradual symptom improvement. The successful management of ASEA requires a multidisciplinary approach involving neurosurgeons, infectious disease specialists, radiologists, and physical therapists. Clinicians should be aware of the possibility of ASEA regardless of systemic symptoms, and early diagnosis and prompt treatment with surgical decompression and appropriate antifungal therapy are imperative for successful management.

## Introduction

Aspergillus spinal epidural abscess (ASEA) is a rare but serious fungal infection near the spinal cord that can cause severe neurologic deficits if left untreated. Typical symptoms include fever, weakness, and back pain but can vary greatly [1]. Although Staphylococcus aureus is the most common cause of spinal epidural abscess (SEA), fungal infections, such as Aspergillus, are extremely rare but more common in the immunocompromised patient. Early diagnosis is crucial to prevent the progression of the disease and treatment involves surgical decompression and antifungal therapy. We present a case of ASEA in an immunocompetent patient, highlighting the challenges in diagnosis and the need for multidisciplinary management of this rare condition. This review also summarizes the existing literature on ASEA, focusing on clinical presentations, diagnostic methods, treatment options, and outcomes.

## Case presentation

An 85-year-old male presented to the office with a six-week history of progressive lower extremity weakness with back pain. Four months prior, the patient was seen for an evaluation and was asymptomatic with normal lower extremity strength and able to stand and ambulate without assistance. His condition worsened since then and he reported having several more falls and developed lower extremity paralysis. He was unable to stand or ambulate independently and now required a wheelchair to ambulate. A Foley catheter was in place due to urinary retention and he required stool softeners for constipation. Medications included tizanidine, tamsulosin, gabapentin, diltiazem, finasteride, omeprazole, losartan, and multivitamins. Past medical history was significant for hypertension, benign prostatic hyperplasia, osteoporosis, degenerative disc disease, colon cancer, and skin cancer. Surgical history included a cervical spine fusion from C4-C7, lumbar spine fusion from L4-L5, decompression and extension fusion from T11-S1 (3 years prior), partial colectomy for cancer, right carpal tunnel release, and right hip replacement.

**Figure 1:**
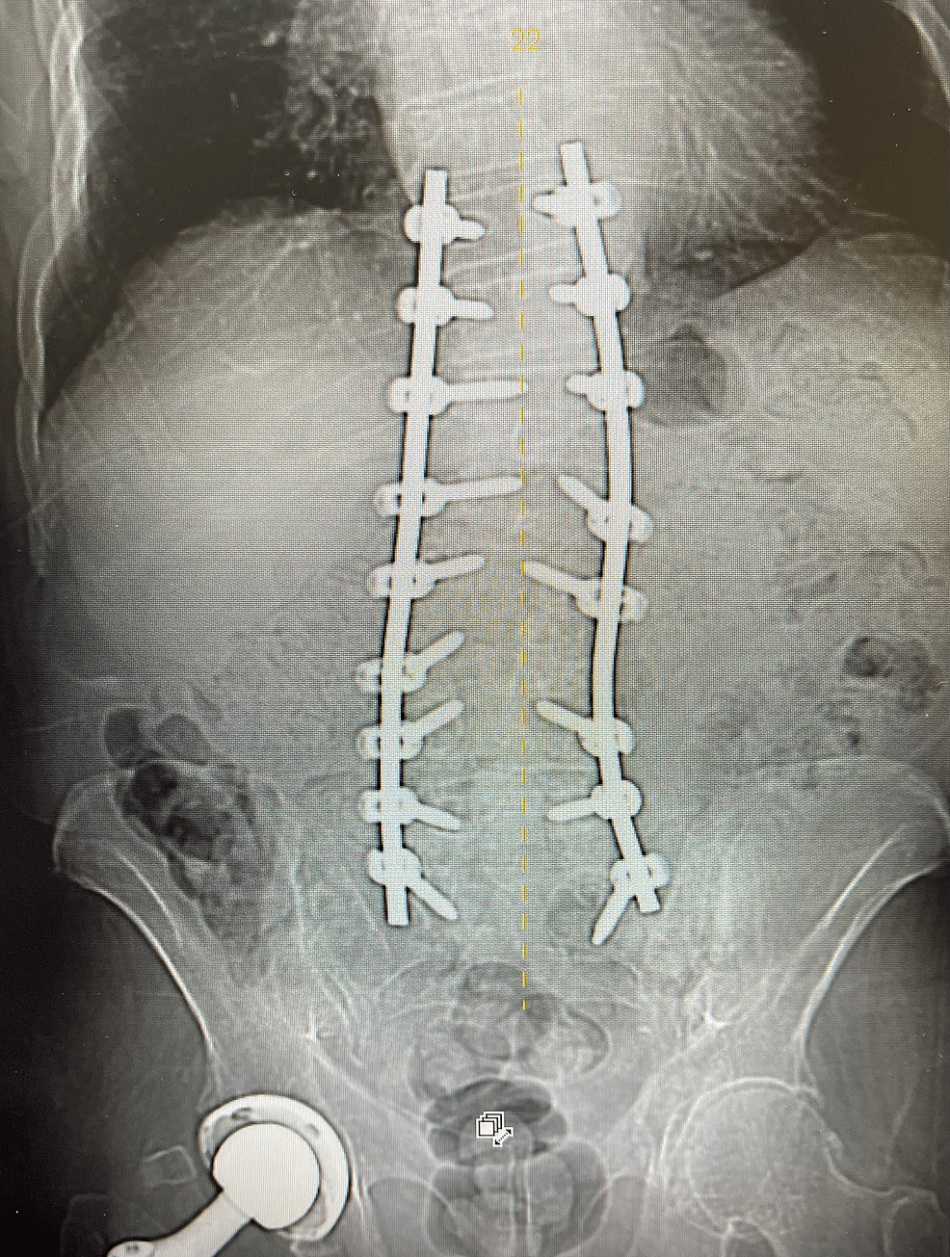
AP view of extensive spinal instrumentation

**Figure 2:**
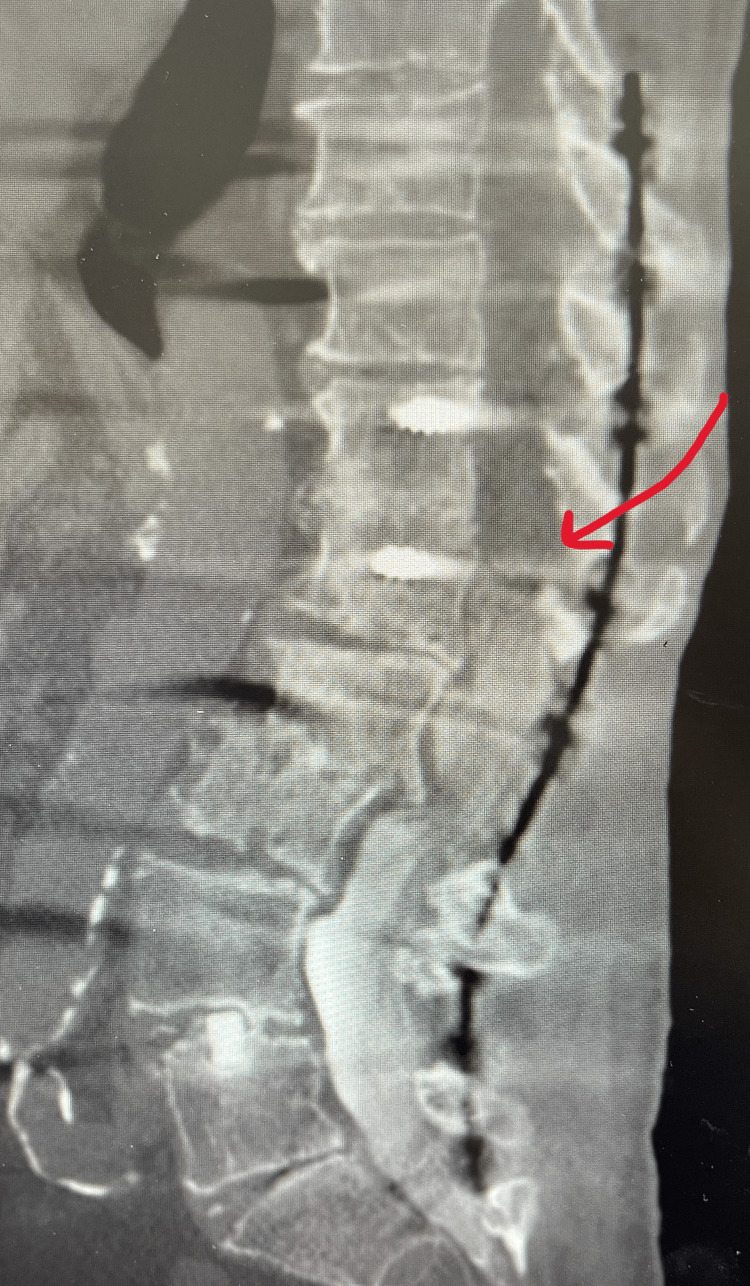
Limited lateral CT myelogram imaging study with IV contrast blockage at L1 (red arrow)

Vitals were within normal limits, along with well-controlled blood pressure. A physical exam revealed mild tenderness over the posterior neck, thoracic back, and lumbar back. Thoracolumbar back incisions remained well-healed. Cranial nerves II through XII were normal. Strength was 4/5 in bilateral upper extremities with mild grip weakness. There was dense paralysis of the right lower extremity with only trace (1/5) movement of the right hip and knee and mild to moderate weakness (4/5) of the left hip, knee, and ankle. Sensation was diminished in stocking distribution in bilateral legs and feet. Reflexes were decreased in knees and ankles. Standard labs, including white blood cell count, CSR, and ESR, were within normal limits. CT myelogram of the thoracolumbar spine showed no spinal canal stenosis above the level of previous fusion. However, the spinal canal near the thoracolumbar junction was not clearly delineated due to artifacts from instrumentation (Figure 1). CT myelogram demonstrated a contrast block at L1, which was suspicious for lumbar spinal stenosis and impingement, myelomalacia, or cord injury (Figure 2).

The patient elected to proceed with spinal exploration at the thoracolumbar junction with repeat laminectomy of T12-L1 and L1-L2. Purulent fluid was found within the epidural space, consistent with an abscess. Gentle suction aspiration easily removed the fluid. There was also a large epidural phlegmon eroding into the thecal sac that was debrided, however, the lower aspect of the defect could not be completely closed due to friable and necrotic dura. The wound was irrigated, vancomycin powder was placed, and a Stratafix suture (Ethicon Inc., Raritan, New Jersey) was used to close the muscular fascia. Subcutaneous tissue and skin were not closed and a wound vacuum sponge was placed in the surgical bed with an occlusive dressing over the vacuum tubing. Specimens consisted of cultures and tissue biopsies and were sent to microbiology. Cultures returned with Aspergillus spp. ID was consulted and recommended starting the patient on cefepime, vancomycin, and voriconazole. The wound vacuum was changed every three days, and the patient was discharged to long-term acute care.

On a one-month follow-up, the patient was taken back to the operating room for debridement and closure of the wound. There was a large amount of hypertrophic granulation tissue that was pink in color and did not appear infected. No gross purulence, erythema, or swelling was noted. The wound was irrigated copiously with Hibiclens solution. The skin and granulation tissue was excised down to the muscular fascia, which was then debrided and irrigated again. All tissue was then closed with sutures and no specimen was forwarded at this time. The patient was then followed every two weeks with progressive resolution of neurologic dysfunction. After one month, the patient was undergoing physical therapy and was able to use a walker to ambulate while his paralysis continued to improve.

## Discussion

Spinal epidural abscess (SEA) comprises two to eight cases per 10,000 hospital admissions [1]. ASEA is an extremely rare type of SEA, with few reported cases in the literature. A search of the PubMed database was conducted using the keywords “aspergillus spinal epidural abscess” and the filter “free full text” applied (Table 1). A total of 12 articles published between 1983 to 2022 were included, accounting for 17 individual cases. The average patient age was 49.5, with a male predominance of 3:2. Immunosuppressed states, including tuberculosis, chronic steroid use, chronic infections, diabetes mellitus, and chronic kidney disease, were present in 12 cases. Some form of trauma was present in four cases and one case presented without any significant comorbid conditions. Diagnostic imaging modalities included MRI in 15 cases, X-ray in one case, and myelogram in one case. Most imaging findings showed a mass with spinal compression. The average number of spinal levels affected was 2.88 with nine cases affecting the thoracic spine, seven cases affecting the lumbar spine, and one case affecting both the thoracic and lumbar spine. All cases were infected with Aspergillus species with A. fumigatus being the most common. Voriconazole was the most commonly used antifungal medication and laminectomy was the preferred surgical intervention. Post-surgical and pharmacological management was used in 14 cases with complete resolution in nine cases, partial resolution in three cases, and death in two cases. Pharmacological management alone was used in three cases of which only one survived.

**Table 1:** A literature review of 17 cases of Aspergillus spinal epidural abscess M: male; F: female; MRI: magnetic resonance imaging

Authors	Year	Age/Sex	Spinal Level	Imaging	Comorbidity	Causative Pathogen	Medications	Operation	Results
Chee et al. [2]	1983	54/M	T3-T4	Myelogram showed blockage of T4 with epidural compression	Asthma, pulmonary tuberculosis	Aspergillus spp.	Amphotericin B, Ketoconazole	Laminectomy of T3-T4	Died 4 months post-surgical and pharmacological management
Saigal et al. [3]	2004	31/F	T10-T11 & T12-L1	MRI showed intradural abscesses causing cord compression	Three lumbar epidural steroid injections for disk herniation	Aspergillus fumigatus	Amphotericin	T9–T11 laminectomy & L4-L5 hemilaminectomy	Resolution of infection with persistent back pain 6 months post-surgical and pharmacological management
Tew et al. [4]	2009	50/M	T2-T9	MRI showed spinal cord compression due to an epidural abscess	Pulmonary tuberculosis, tuberculous bronchiectasis, right middle lobe lobectomy	Aspergillus fumigatus	Voriconazole	T2-T8 laminectomy	Died 2 weeks post-surgical and pharmacological management
Batra et al. [5]	2011	45/M	L3-S1	MRI showed a multilocular extradural collection	None	Aspergillus fumigatus	Itraconazole	Laminectomy of L3-L5 and surgical decompression	Complete resolution 1 week post-surgical and pharmacological management
Jiang et al. [6]	2013	40/F	T1-T3	MRI showed anterior and posterior epidural abscesses	Lung fungal granuloma and brain cysticercosis	Aspergillus nidulans	Voriconazole	Laminectomy of T1–T3 and debridement	Complete resolution 1 year post-surgical and pharmacological management
Raj et al. [7]	2013	45/F	L5-S1	MRI showed anterior epidural granulation tissue/abscess	Diabetes	Aspergillus fumigatus	Itraconazole	Laminectomy of L5-S1	Complete resolution 9 months post-surgical and pharmacological management
Sathyapalan et al. [8]	2016	35/F	T5-T9	MRI showed an epidural mass	Chronic oral steroid use for pulmonary sarcoidosis	Aspergillus fumigatus	Voriconazole	T5-T8 laminectomy and debridement	Complete resolution 2 years post-surgical and pharmacological management
Shweikeh et al. [9]	2018	58/F	L4-S1	MRI showed an epidural abscess	Chronic oral steroid use	Aspergillus spp.	Voriconazole and Micafungin	None	Complete resolution 3 months post pharmacological management
Takagi et al. [10]	2019	74/M	T11-T12	MRI showed cord compression and epidural abscess	Left abdominal stab wound	Aspergillus terreus	Voriconazole	Partial laminectomy at T11 and posterior fusion at T9-L2	Complete resolution 2 years post-surgical and pharmacological management
Tavakoli et al. [11]	2020	10/M	T4-T5	X-ray showed destructive soft tissue density mass lesion	Chronic granulomatous disease	Aspergillus nidulans	Amphotericin B, Caspofungin, Voriconazole, and Interferon-gamma	Right parietal ventriculoperitoneal shunt	Died within 1 year post pharmacological management
Dai et al. [12]	2020	67/M	T3-T5	MRI	Lung cancer surgery, pulmonary aspergillosis	Aspergillus fumigatus	Voriconazole	None	Complete resolution post pharmacological management
		68/M	T12-L2	MRI	Chronic steroid use	Aspergillus fumigatus	Voriconazole	Laminectomy, debridement, decompression, Instrumentation	Resolved infection with residual left limb numbness post-surgical and pharmacological management
		50/F	L3-L4	MRI	Renal failure, hemodialysis	Aspergillus fumigatus	Voriconazole	Laminectomy, debridement, decompression, Instrumentation	Resolved infection with residual lumbar pain post-surgical and pharmacological management
		48/M	L4-L5	MRI	Spine surgery	Aspergillus fumigatus	Voriconazole	Laminectomy, debridement, decompression, Instrumentation	Complete resolution post-surgical and pharmacological management
		43/M	L4-L5	MRI	Spine surgery	Aspergillus niger	Voriconazole	Laminectomy, debridement, decompression, Instrumentation	Complete resolution post-surgical and pharmacological management
		66/M	L2-L3	MRI	Spine surgery	Aspergillus spp.	Voriconazole	Laminectomy, debridement, decompression, Instrumentation	Complete resolution post-surgical and pharmacological management
Rashid et al. [13]	2022	58/F	T11-T12	MRI showed epidural abscess with discitis and osteomyelitis at T11–T12	Diabetes	Aspergillus spp.	Voriconazole, Rifampicin, Pyrazinamide, Ethambutol, Isoniazid, and Pyridoxine	Partial T11–T12 laminectomy	Complete resolution post-surgical and pharmacological management

This opportunistic infection is most commonly seen in immunocompromised patients, including those on corticosteroid therapy [13-15]. The typical clinical presentation of ASEA includes nonspecific symptoms such as fever, back pain, weakness, and paresis [15]. However, our patient only complained of weakness and paresis and had no overt systemic signs of infection (e.g., fever or leukocytosis). He had no recent or past steroid use and history revealed no relevant comorbid conditions that would indicate an immunocompromised status. Based on our literature review, over 70% of patients with ASEA had relevant comorbidity making them immunocompromised.

MRI is the imaging modality of choice for diagnosing ASEA but in our patient’s case, the initial MRI was limited due to extensive artifact from spinal instrumentation. The presence of metal-related artifacts, such as that in our patient, is known to obscure relevant anatomical structures and disease on conventional CT and MRI [16]. Nearly 90% of the cases in our literature review utilized MRI to diagnose ASEA. Conversely, our patient was diagnosed using CT with contrast and although it was still challenging to interpret, rather than prolong diagnosis and treatment for better imaging studies, the patient was offered surgical lumbar exploration.

Emergent evacuation of purulent fluid is the mainstay of treatment, which was consistent with our review, as over 80% of the cases performed surgical laminectomy with a washout of any purulent fluid. Similarly, empiric antibiotics for the most likely causative organism, i.e., Staphylococcus aureus are used [17]. Hence, after initial debridement, vancomycin was utilized but after receiving microbiology results, antimicrobials were tailored toward Aspergillus spp. with voriconazole. A randomized trial conducted by Herbrecht et al., found that voriconazole had higher efficacy in treating primary invasive aspergillosis and fewer adverse side effects than amphotericin B [18]. Furthermore, our review found that voriconazole has become the mainstay antifungal in ASEA, as it was used in over 70% of cases.

## Conclusions

This case report and literature review highlights the rarity and clinical challenges posed by ASEA in an immunocompetent patient. Immunocompromised states were prevalent among nearly all affected patients. Regardless, timely diagnosis, operative laminectomy, and targeted antifungal therapy, predominantly using voriconazole, were vital in achieving favorable outcomes. However, artifacts from extensive spinal instrumentation hindered imaging accuracy, which calls for future investigations into alternative imaging modalities and increased suspicion for SEA even in the absence of typical symptoms and laboratory findings.
